# Nonalcoholic Steatohepatitis and Hepatocellular Carcinoma: Brazilian Survey

**DOI:** 10.6061/clinics/2016(08)02

**Published:** 2016-08

**Authors:** Venancio Avancini Ferreira Alves, Luiz Augusto Carneiro D’Albuquerque

**Affiliations:** I Hospital das Clínicas da Faculdade de Medicina da Universidade de São Paulo, Anatomia Patológica, São Paulo/SP, Brazil; II Faculdade de Medicina da Universidade de São Paulo, Gastroenterologia, São Paulo/SP, Brazil

The incidence of hepatocellular carcinoma (HCC) has increased in recent years, not only in Asia and in Africa, where it is the second leading cancer, but also in Europe and the USA 1. Most HCC cases occur in patients with advanced chronic liver disease related to chronic hepatitis C virus (HCV) infection, chronic hepatitis B (HBV) infection or alcohol abuse. In the last decade, an increasing number of publications have reported an association between nonalcoholic fatty liver disease (NAFLD) and HCC 2.

We recently reported on a series of 42 patients with HCC related to either NAFLD or to cryptogenic cirrhosis and showed that the patients were older, predominantly male (62%) and commonly (81%) had metabolic syndrome risk factors, such as obesity, diabetes, hypertension, or dyslipidemia. We found evidence of cirrhosis in 38 patients (90%), either through liver biopsy or based on clinical signs / symptoms. The diagnosis of HCC was achieved through a screening program in 55% of the 42 patients 3.

In a robust study assessing the epidemiology and surveillance of patients with HCC in the USA, Younossi et al. found that the prevalence of HCC in patients with NAFLD increased by 9% over a six-year of the evaluation period (2004-2009). They also reported that NAFLD increased the risk of 1-year mortality and that it will be a major cause of HCC in the USA 2.

In our Liver Transplant Unit, HCFMUSP, we transplanted 123 patients with HCC over the past 4 years. Six patients (4.9%) had a previous diagnosis of NAFLD. Seven of the cases (5.7%) had cryptogenic cirrhosis. In these cases, the average BMI was 26.3, but 25% had a BMI higher than 28 and 10% had a BMI over 32. Diabetes was present in 29.3% of the patients. These data contrast with reference to NASH, as only 1.9% of a previous cohort of patients with HCC also presented with NASH (our unpublished data).

In a recent issue of CLINICS, Cotrim et al. 4 reported on a Brazilian survey of 110 HCC cases with NAFLD from nine hepatology units in six Brazilian states. Similar to the findings from Younossi et al. 2, these patients were older (mean age=67 years), and 65.5% were male. Obesity was observed in 52.7% of the cases, diabetes in 73.6%, dyslipidemia in 41.0%, hypertension in 60% and metabolic syndrome in 57.2%.

The nature of a national survey imparts some limitations. For example, there is heterogeneity in the methods used to acquire clinical and laboratorial data, there is no standardization of therapeutic procedures and there is no patient follow-up. Even so, this survey was successful in providing insight into the growing prevalence of patients with NASH in Brazil. It also highlighted the need to standardize clinical data regarding not only the potential for progression to cirrhosis but also the actual possibility of development of HCC, including for patients with NASH without fully developed cirrhosis. These data, aligned with our own experience, have caused our group to debate the need to survey patients with NASH even before cirrhosis is fully developed. Our main concern in this regard is the excess workload for ultrasonographists, whereas performing other imaging methods would be excessively expensive.

In a study reported by Cotrim et al. 4, histological diagnosis of HCC was performed in 47.2% of patients. Out of 48 cases where non-tumoral samples were available for histopathological study, NASH-related cirrhosis was diagnosed in 32 cases (66.7%), NASH + fibrosis (grade 1-3) was diagnosed in 14 cases (29.1%) and NASH without fibrosis was diagnosed in 2 cases (4.2%). Brazilian clinicians, radiologists and surgeons have also biopsied liver nodules and non-neoplastic livers, which has further improved understanding of the heterogeneity of the clinical and pathological settings that foster HCC development. Careful follow-up of patients also enables better selection of high-risk patients, who should undergo surveillance procedures, as recently reported by Vezozzo et al. 5. As in recent years, liver biopsy has become highly valued for addressing major questions related to hepatocarcinogenesis 6,7, more collaborative studies involving multidisciplinary Brazilian study groups to identify further associations between epidemiological and clinical variables and morphological and molecular biomarkers are expected in the future.
